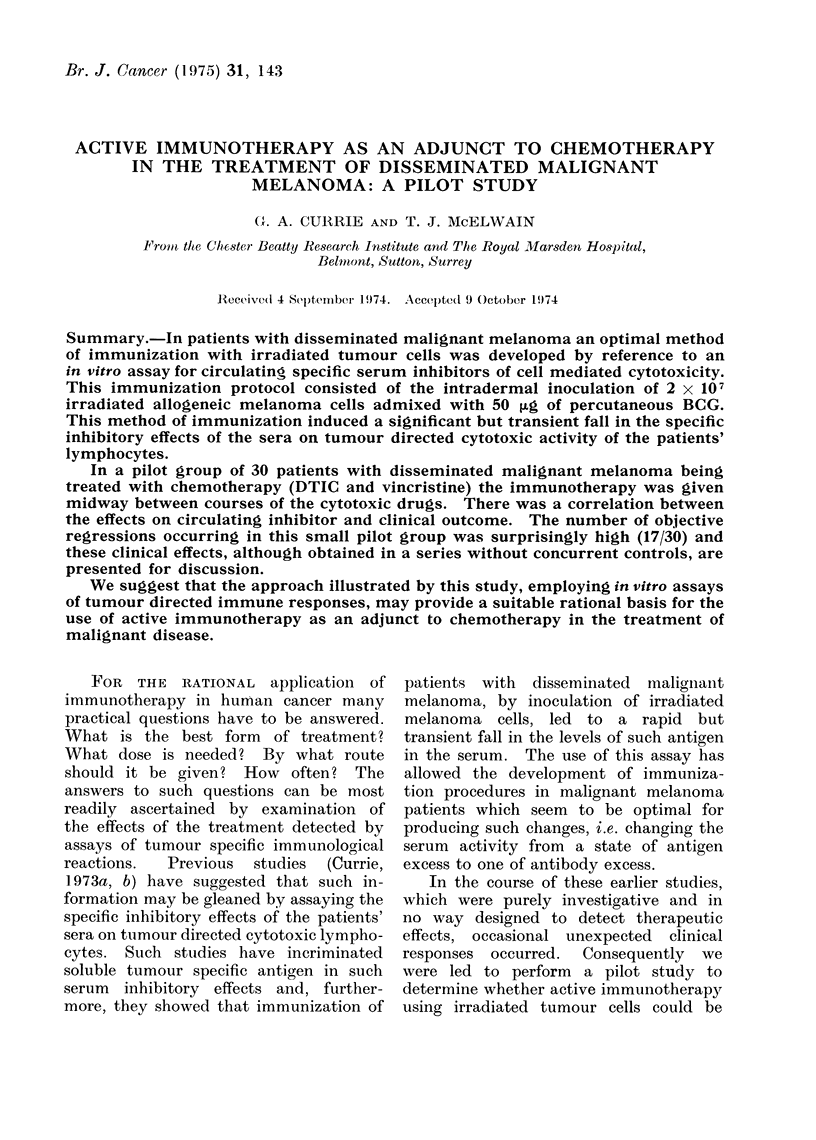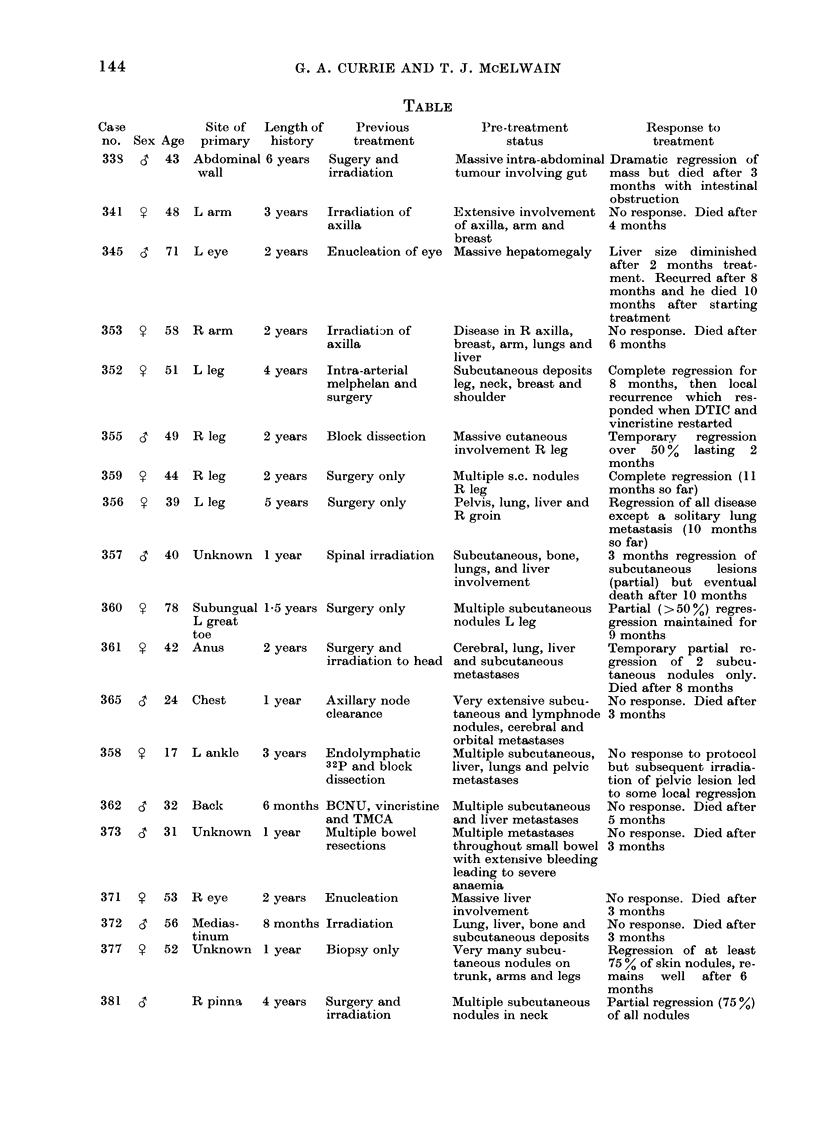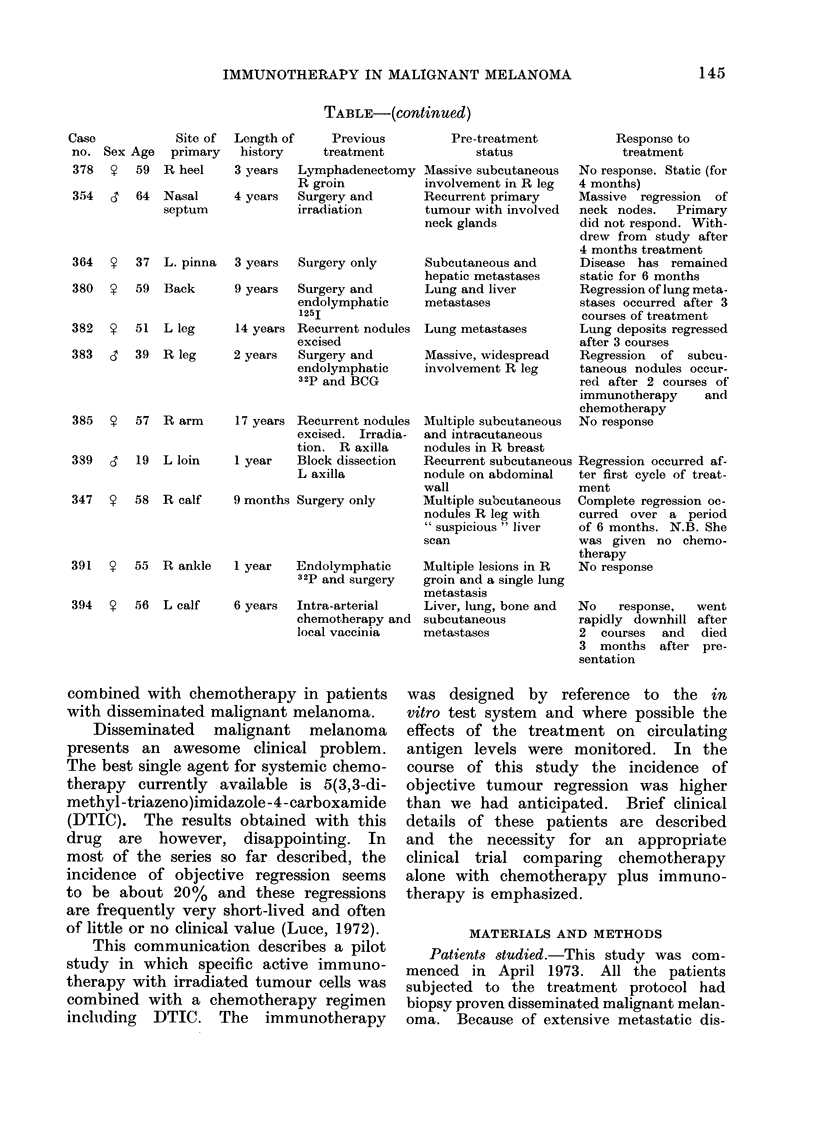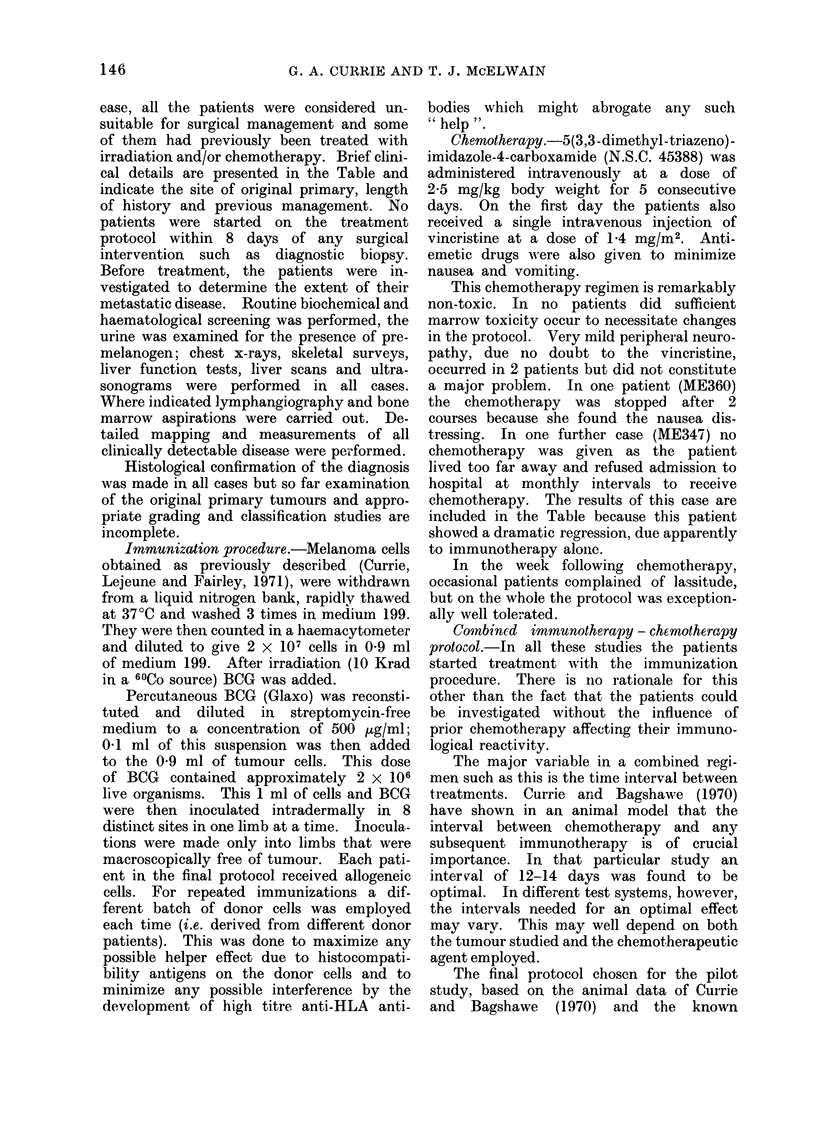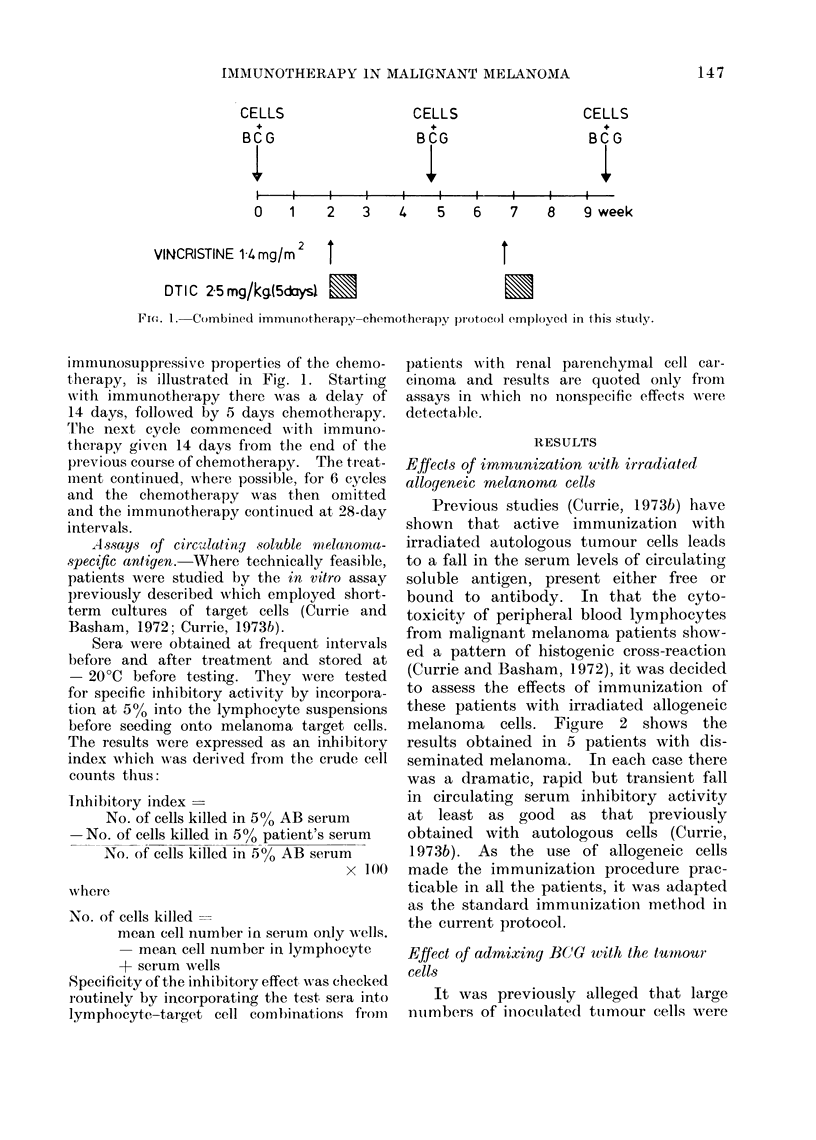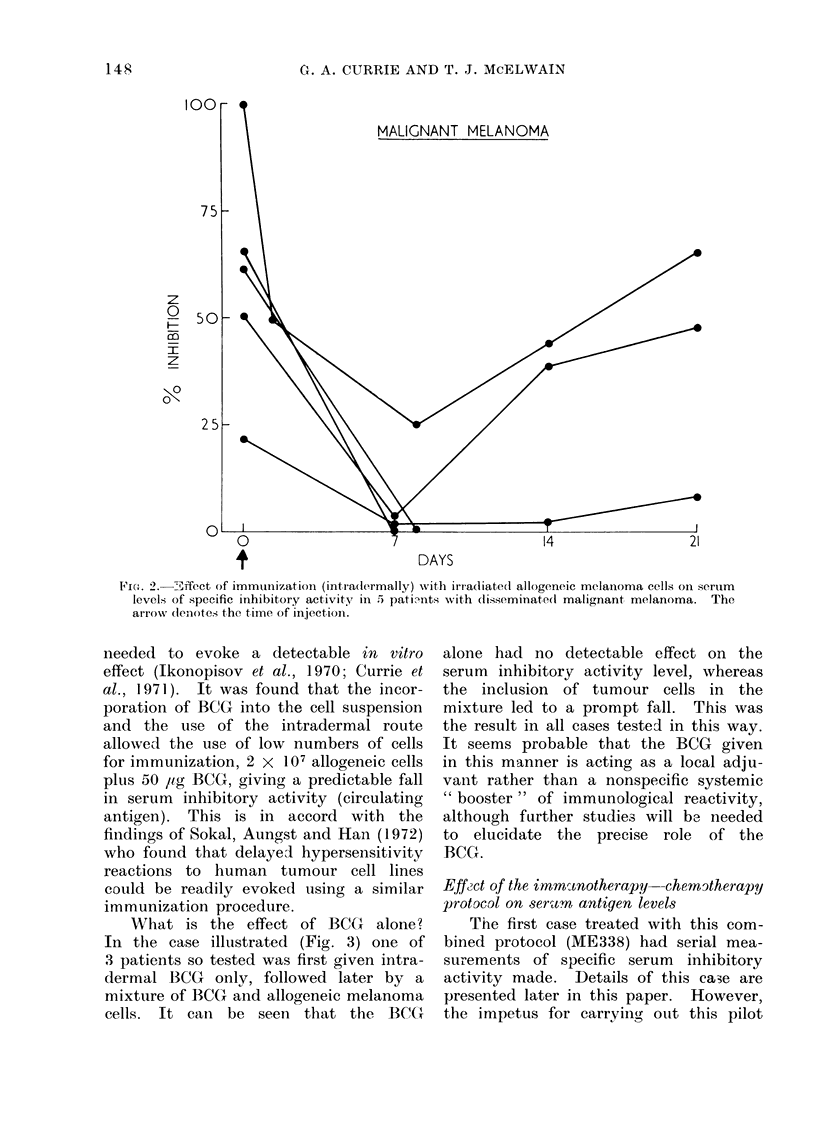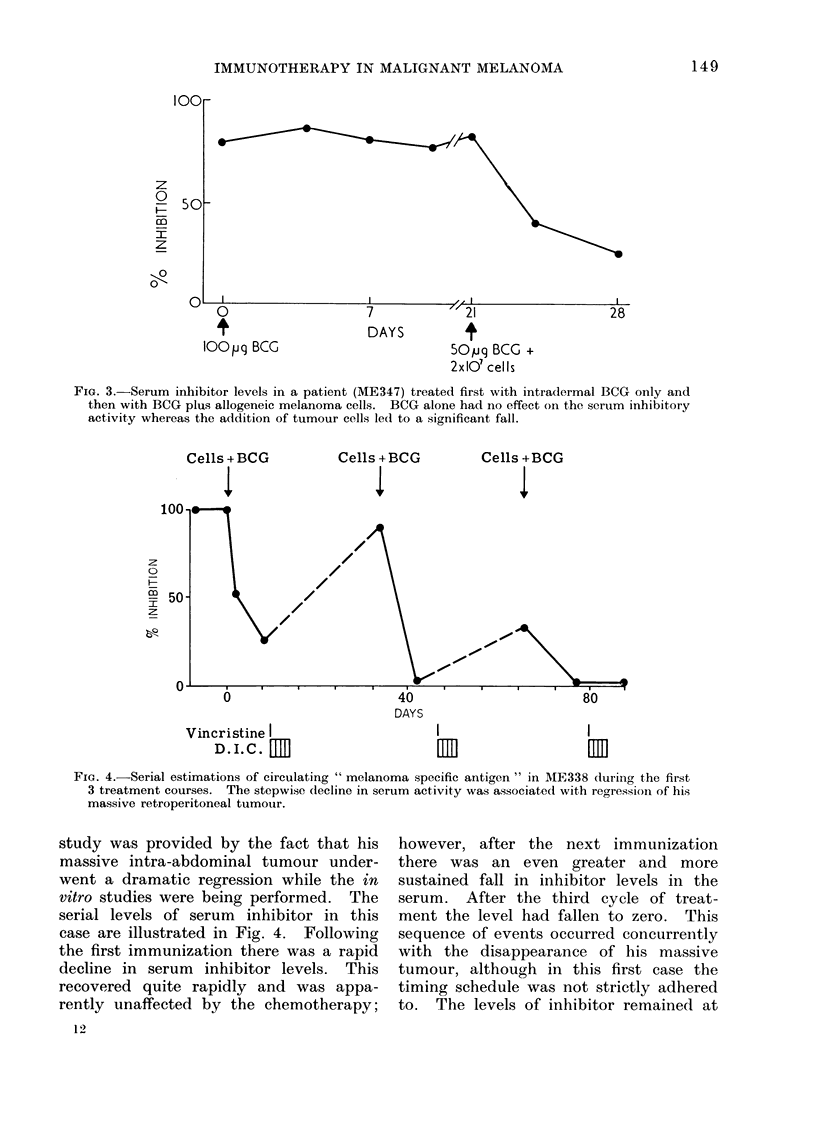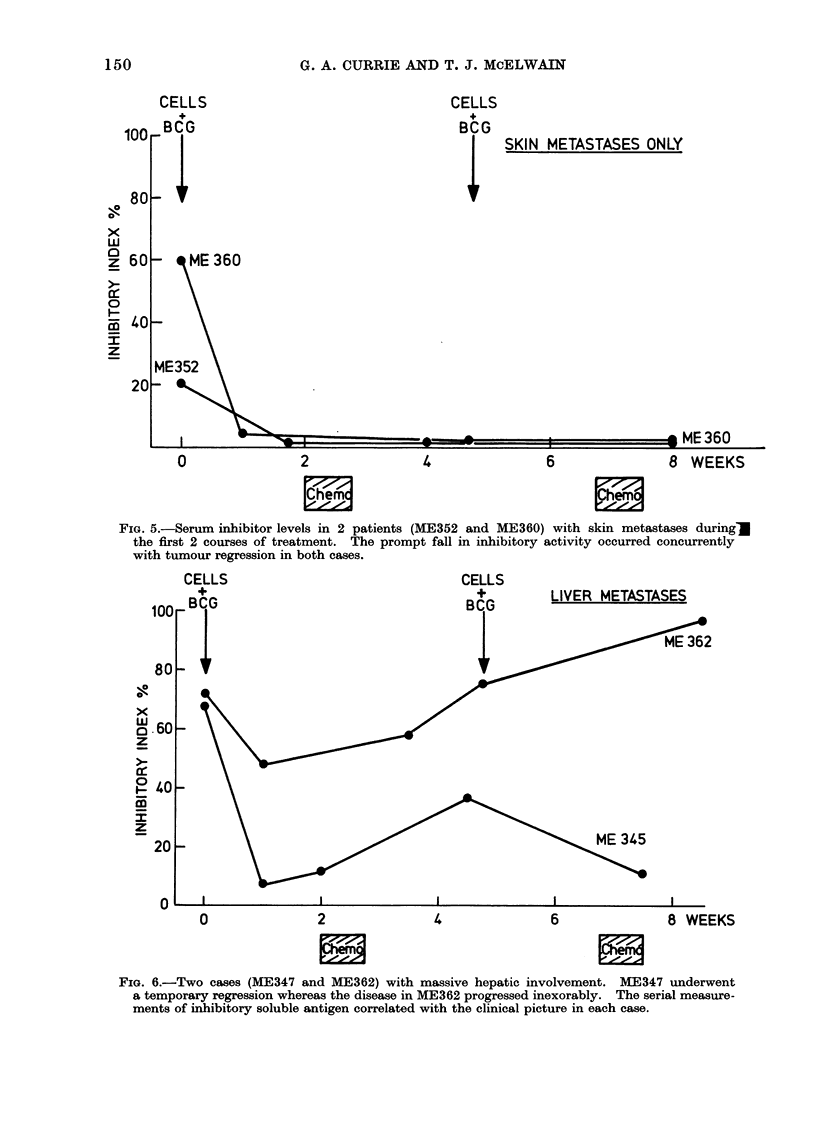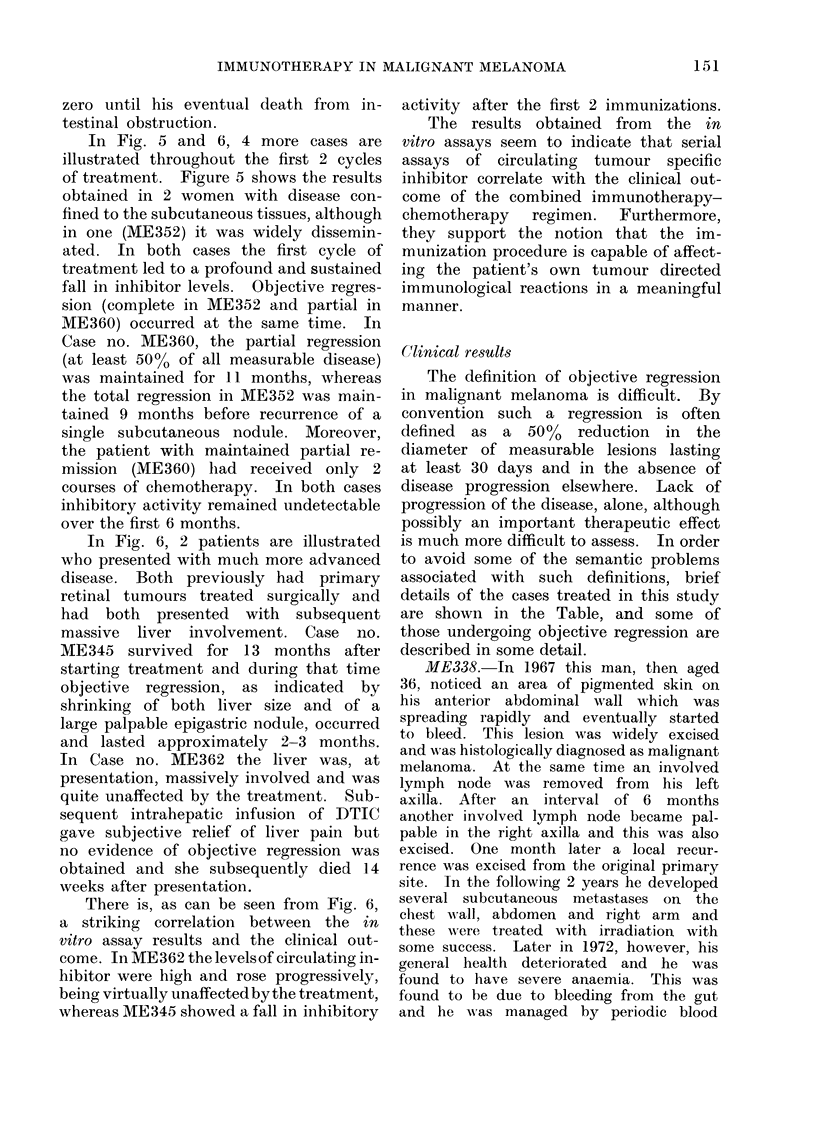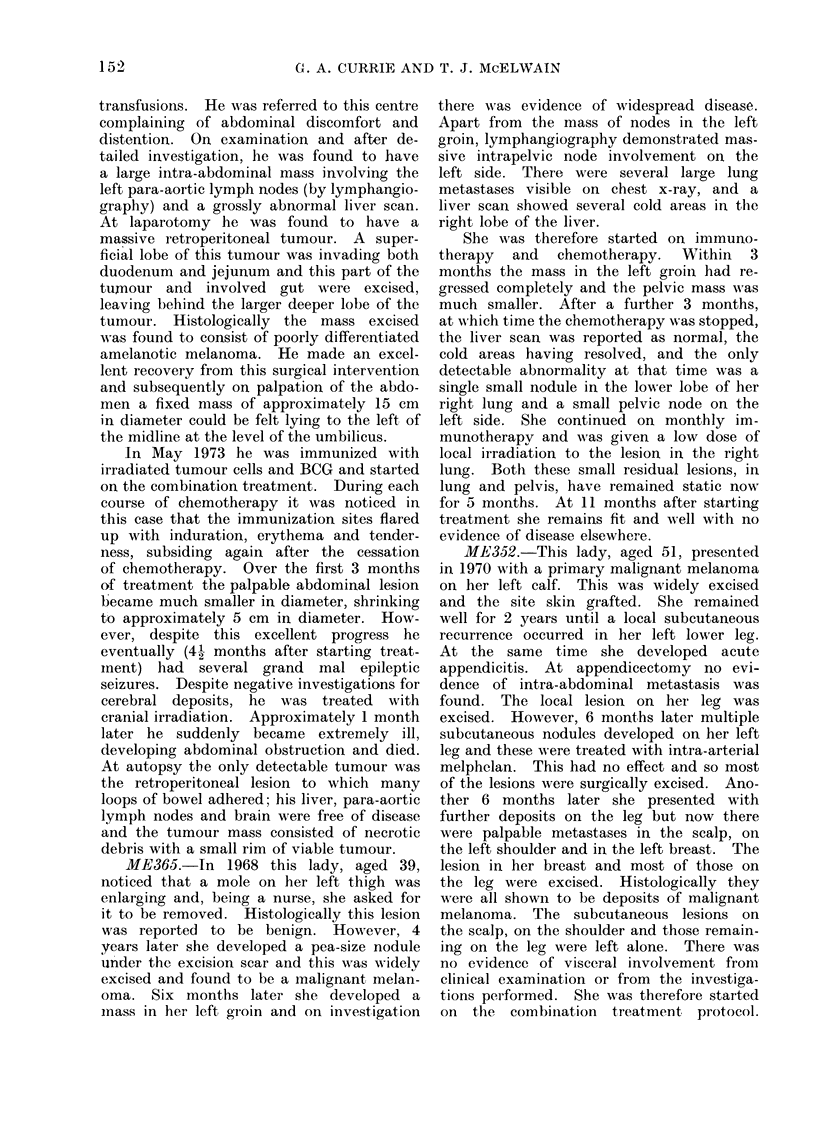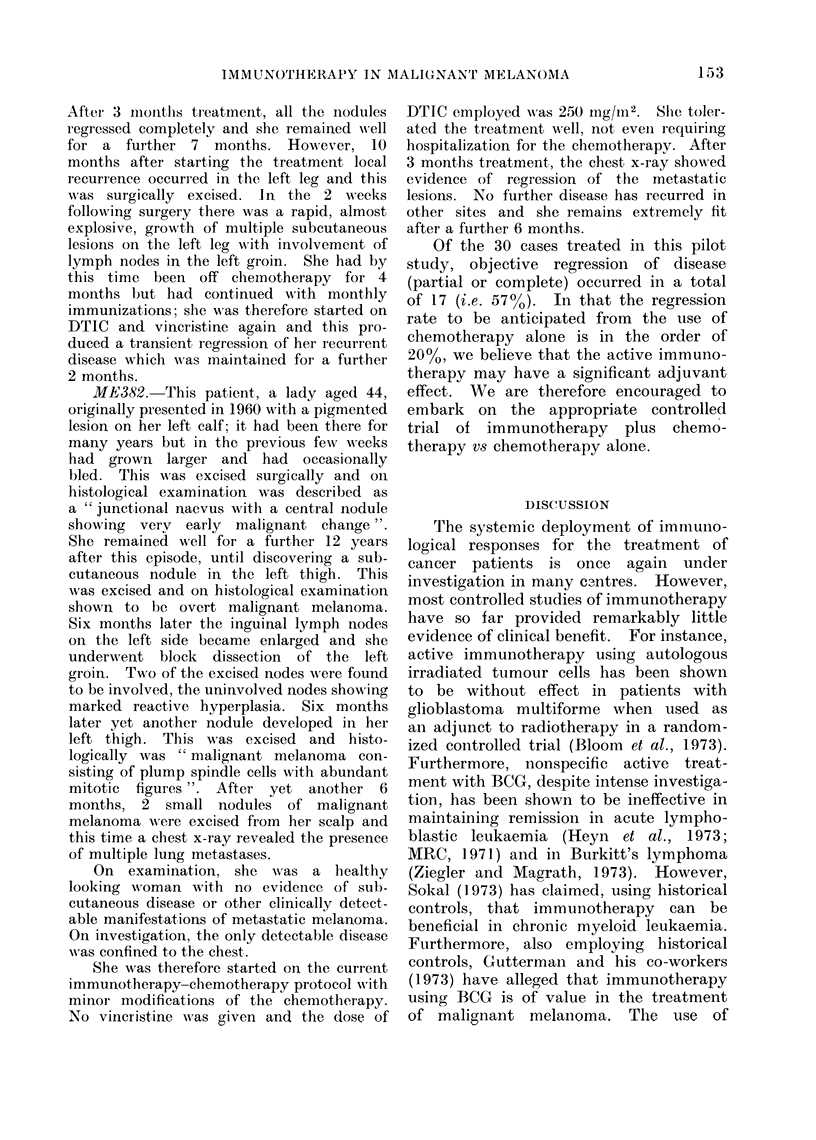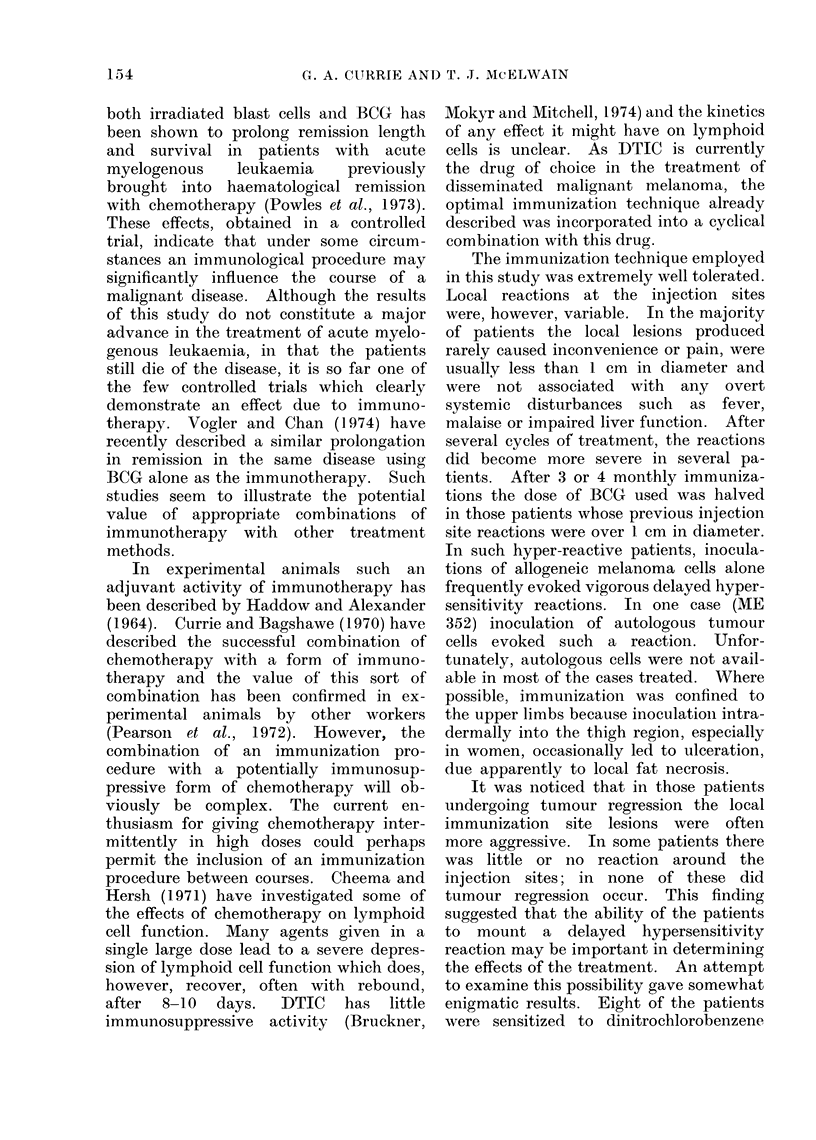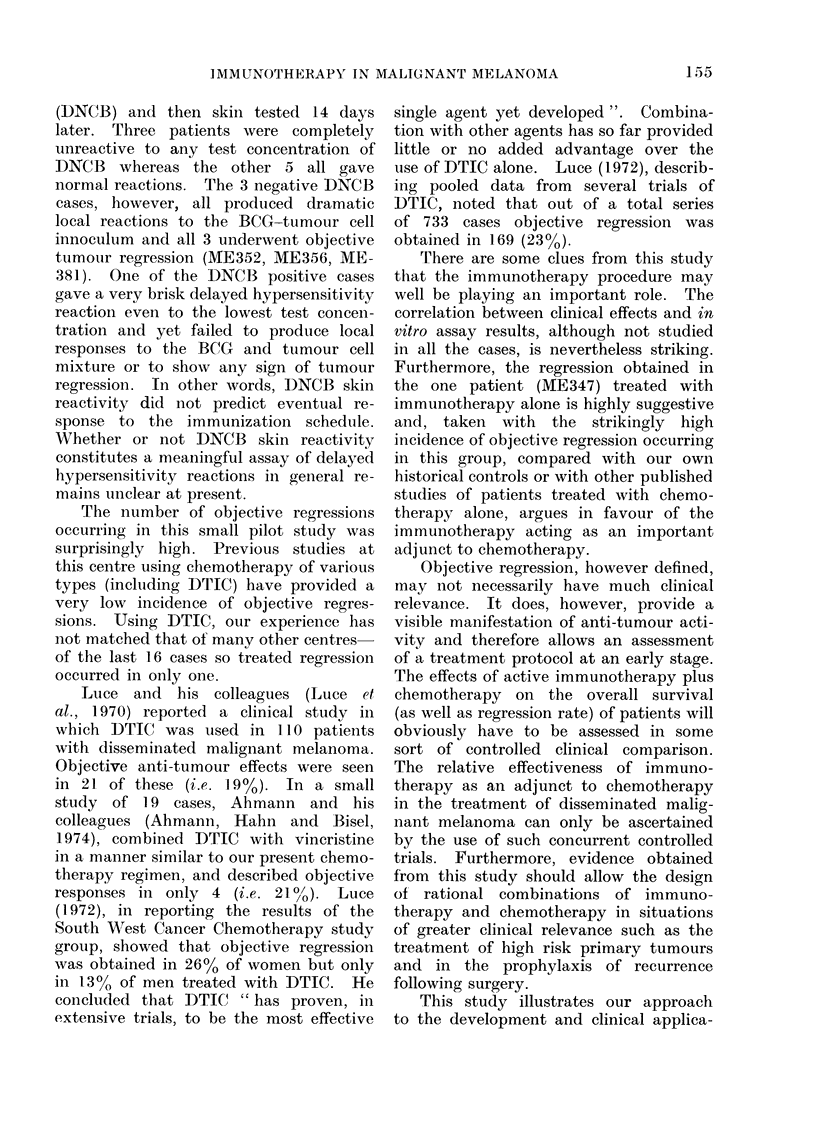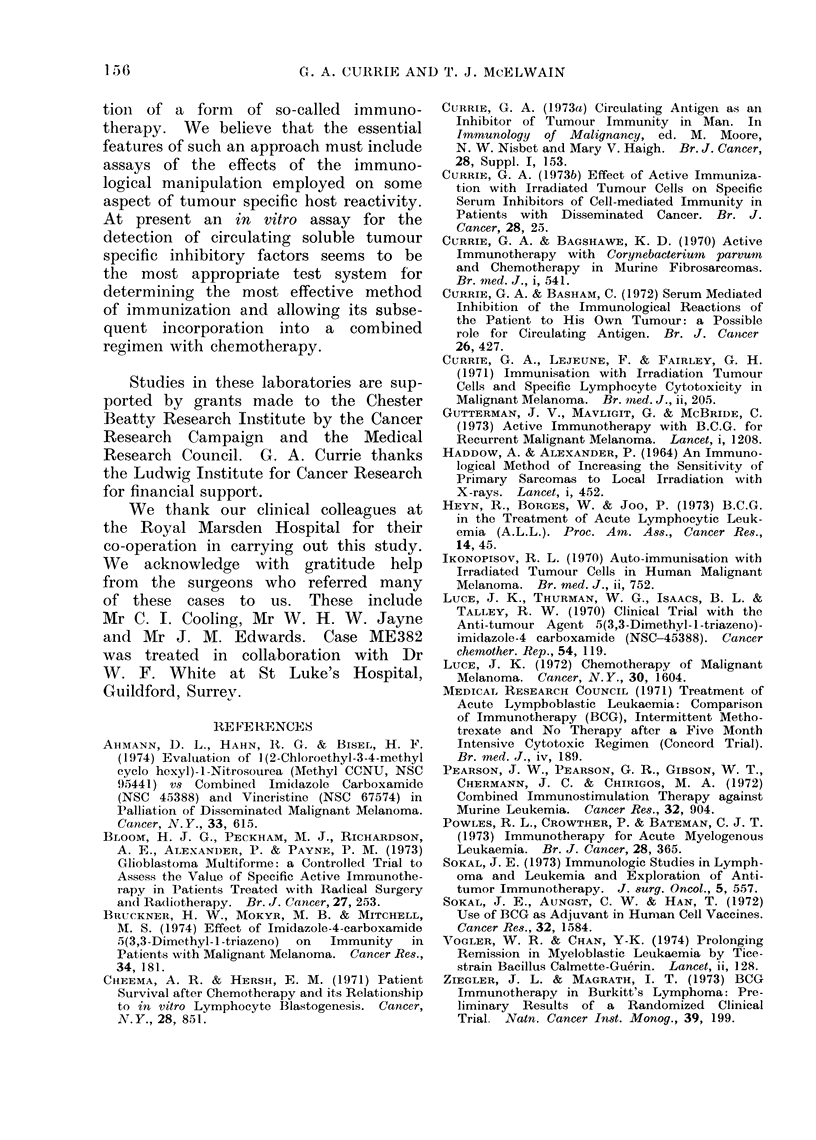# Active immunotherapy as an adjunct to chemotherapy in the treatment of disseminated malignant melanoma: a pilot study.

**DOI:** 10.1038/bjc.1975.19

**Published:** 1975-02

**Authors:** G. A. Currie, T. J. McElwain

## Abstract

In patients with disseminated malignant melanoma an optimal method of immunization with irradiated tumour cells was developed by reference to an in vitro assay for circulating specific serum inhibitors of cell mediated cytotoxicity. This immunization protocol consisted of the intradermal inoculation of 2 times 10(7) irradiated allogeneic melanoma cells admixed with 50 mug of percutaneous BCG. This method of immunization induced a significant but transient fall in the specific inhibitory effects of the sera on tumour directed cytotoxic activity of the patients' lymphocytes. In a pilot group of 30 patients with disseminated malignant melanoma being treated with chemotherapy (DTIC and vincristine) the immunotherapy was given midway between courses of the cytotoxic drugs. There was a correlation between the effects on circulating inhibitor and clinical outcome. The number of objective regressions occurring in this small pilot group was surprisingly high (17/30) and these clinical effects, although obtained in a series without concurrent controls, are presented for discussion. We suggest that the approach illustrated by this study, employing in vitro assays of tumour directed immune responses, may provide a suitable rational basis for the use of active immunotherapy as an adjunct to chemotherapy in the treatment of malignant disease.


*Br. J. Cancer* (1975) **31**, 143

### ACTIVE IMMUNOTHERAPY AS AN ADJUNCT TO CHEMOTHERAPY IN THE TREATMENT OF DISSEMINATED MALIGNANT MELANOMA: A PILOT STUDY

G. A. CURRIE AND T. J. McELWAIN

*From the Chester Beatty Research Institute and The Royal Marsden Hospital, Belmont, Sutton, Surrey*

Received 4 September 1974. Accepted 9 October 1974

**Summary.—In patients with disseminated malignant melanoma an optimal method of immunization with irradiated tumour cells was developed by reference to an *in vitro* assay for circulating specific serum inhibitors of cell mediated cytotoxicity. This immunization protocol consisted of the intradermal inoculation of 2 × 10⁷ irradiated allogeneic melanoma cells admixed with 50 µg of percutaneous BCG. This method of immunization induced a significant but transient fall in the specific inhibitory effects of the sera on tumour directed cytotoxic activity of the patients' lymphocytes.**

**In a pilot group of 30 patients with disseminated malignant melanoma being treated with chemotherapy (DTIC and vincristine) the immunotherapy was given midway between courses of the cytotoxic drugs. There was a correlation between the effects on circulating inhibitor and clinical outcome. The number of objective regressions occurring in this small pilot group was surprisingly high (17/30) and these clinical effects, although obtained in a series without concurrent controls, are presented for discussion.**

**We suggest that the approach illustrated by this study, employing *in vitro* assays of tumour directed immune responses, may provide a suitable rational basis for the use of active immunotherapy as an adjunct to chemotherapy in the treatment of malignant disease.**

FOR THE RATIONAL application of immunotherapy in human cancer many practical questions have to be answered. What is the best form of treatment? What dose is needed? By what route should it be given? How often? The answers to such questions can be most readily ascertained by examination of the effects of the treatment detected by assays of tumour specific immunological reactions. Previous studies (Currie, 1973*a*, *b*) have suggested that such information may be gleaned by assaying the specific inhibitory effects of the patients' sera on tumour directed cytotoxic lymphocytes. Such studies have incriminated soluble tumour specific antigen in such serum inhibitory effects and, furthermore, they showed that immunization of patients with disseminated malignant melanoma, by inoculation of irradiated melanoma cells, led to a rapid but transient fall in the levels of such antigen in the serum. The use of this assay has allowed the development of immunization procedures in malignant melanoma patients which seem to be optimal for producing such changes, *i.e.* changing the serum activity from a state of antigen excess to one of antibody excess.

In the course of these earlier studies, which were purely investigative and in no way designed to detect therapeutic effects, occasional unexpected clinical responses occurred. Consequently we were led to perform a pilot study to determine whether active immunotherapy using irradiated tumour cells could be

TABLE

| Case no. | Sex | Age | Site of primary | Length of history | Previous treatment | Pre-treatment status | Response to treatment |
|---|---|---|---|---|---|---|---|
| 338 | ♂ | 43 | Abdominal wall | 6 years | Sugery and irradiation | Massive intra-abdominal tumour involving gut | Dramatic regression of mass but died after 3 months with intestinal obstruction |
| 341 | ♀ | 48 | L arm | 3 years | Irradiation of axilla | Extensive involvement of axilla, arm and breast | No response. Died after 4 months |
| 345 | ♂ | 71 | L eye | 2 years | Enucleation of eye | Massive hepatomegaly | Liver size diminished after 2 months treatment. Recurred after 8 months and he died 10 months after starting treatment |
| 353 | ♀ | 58 | R arm | 2 years | Irradiation of axilla | Disease in R axilla, breast, arm, lungs and liver | No response. Died after 6 months |
| 352 | ♀ | 51 | L leg | 4 years | Intra-arterial melphelan and surgery | Subcutaneous deposits leg, neck, breast and shoulder | Complete regression for 8 months, then local recurrence which responded when DTIC and vincristine restarted |
| 355 | ♂ | 49 | R leg | 2 years | Block dissection | Massive cutaneous involvement R leg | Temporary regression over 50% lasting 2 months |
| 359 | ♀ | 44 | R leg | 2 years | Surgery only | Multiple s.c. nodules R leg | Complete regression (11 months so far) |
| 356 | ♀ | 39 | L leg | 5 years | Surgery only | Pelvis, lung, liver and R groin | Regression of all disease except a solitary lung metastasis (10 months so far) |
| 357 | ♂ | 40 | Unknown | 1 year | Spinal irradiation | Subcutaneous, bone, lungs, and liver involvement | 3 months regression of subcutaneous lesions (partial) but eventual death after 10 months |
| 360 | ♀ | 78 | Subungual L great toe | 1·5 years | Surgery only | Multiple subcutaneous nodules L leg | Partial (>50%) regression maintained for 9 months |
| 361 | ♀ | 42 | Anus | 2 years | Surgery and irradiation to head | Cerebral, lung, liver and subcutaneous metastases | Temporary partial regression of 2 subcutaneous nodules only. Died after 8 months |
| 365 | ♂ | 24 | Chest | 1 year | Axillary node clearance | Very extensive subcutaneous and lymphnode nodules, cerebral and orbital metastases | No response. Died after 3 months |
| 358 | ♀ | 17 | L ankle | 3 years | Endolymphatic $^{32}P$ and block dissection | Multiple subcutaneous, liver, lungs and pelvic metastases | No response to protocol but subsequent irradiation of pelvic lesion led to some local regression |
| 362 | ♂ | 32 | Back | 6 months | BCNU, vincristine and TMCA | Multiple subcutaneous and liver metastases | No response. Died after 5 months |
| 373 | ♂ | 31 | Unknown | 1 year | Multiple bowel resections | Multiple metastases throughout small bowel with extensive bleeding leading to severe anaemia | No response. Died after 3 months |
| 371 | ♀ | 53 | R eye | 2 years | Enucleation | Massive liver involvement | No response. Died after 3 months |
| 372 | ♂ | 56 | Mediastinum | 8 months | Irradiation | Lung, liver, bone and subcutaneous deposits | No response. Died after 3 months |
| 377 | ♀ | 52 | Unknown | 1 year | Biopsy only | Very many subcutaneous nodules on trunk, arms and legs | Regression of at least 75% of skin nodules, remains well after 6 months |
| 381 | ♂ | | R pinna | 4 years | Surgery and irradiation | Multiple subcutaneous nodules in neck | Partial regression (75%) of all nodules |

TABLE—(*continued*)

| Case no. | Sex | Age | Site of primary | Length of history | Previous treatment | Pre-treatment status | Response to treatment |
|---|---|---|---|---|---|---|---|
| 378 | ♀ | 59 | R heel | 3 years | Lymphadenectomy R groin | Massive subcutaneous involvement in R leg | No response. Static (for 4 months) |
| 354 | ♂ | 64 | Nasal septum | 4 years | Surgery and irradiation | Recurrent primary tumour with involved neck glands | Massive regression of neck nodes. Primary did not respond. Withdrew from study after 4 months treatment |
| 364 | ♀ | 37 | L. pinna | 3 years | Surgery only | Subcutaneous and hepatic metastases | Disease has remained static for 6 months |
| 380 | ♀ | 59 | Back | 9 years | Surgery and endolymphatic $^{125}I$ | Lung and liver metastases | Regression of lung metastases occurred after 3 courses of treatment |
| 382 | ♀ | 51 | L leg | 14 years | Recurrent nodules excised | Lung metastases | Lung deposits regressed after 3 courses |
| 383 | ♂ | 39 | R leg | 2 years | Surgery and endolymphatic $^{32}P$ and BCG | Massive, widespread involvement R leg | Regression of subcutaneous nodules occurred after 2 courses of immunotherapy and chemotherapy |
| 385 | ♀ | 57 | R arm | 17 years | Recurrent nodules excised. Irradiation. R axilla | Multiple subcutaneous and intracutaneous nodules in R breast | No response |
| 339 | ♂ | 19 | L loin | 1 year | Block dissection L axilla | Recurrent subcutaneous nodule on abdominal wall | Regression occurred after first cycle of treatment |
| 347 | ♀ | 58 | R calf | 9 months | Surgery only | Multiple subcutaneous nodules R leg with " suspicious " liver scan | Complete regression occurred over a period of 6 months. N.B. She was given no chemotherapy |
| 391 | ♀ | 55 | R ankle | 1 year | Endolymphatic $^{32}P$ and surgery | Multiple lesions in R groin and a single lung metastasis | No response |
| 394 | ♀ | 56 | L calf | 6 years | Intra-arterial chemotherapy and local vaccinia | Liver, lung, bone and subcutaneous metastases | No response, went rapidly downhill after 2 courses and died 3 months after presentation |

combined with chemotherapy in patients with disseminated malignant melanoma.

Disseminated malignant melanoma presents an awesome clinical problem. The best single agent for systemic chemotherapy currently available is 5(3,3-dimethyl-triazeno)imidazole-4-carboxamide (DTIC). The results obtained with this drug are however, disappointing. In most of the series so far described, the incidence of objective regression seems to be about 20% and these regressions are frequently very short-lived and often of little or no clinical value (Luce, 1972).

This communication describes a pilot study in which specific active immunotherapy with irradiated tumour cells was combined with a chemotherapy regimen including DTIC. The immunotherapy was designed by reference to the *in vitro* test system and where possible the effects of the treatment on circulating antigen levels were monitored. In the course of this study the incidence of objective tumour regression was higher than we had anticipated. Brief clinical details of these patients are described and the necessity for an appropriate clinical trial comparing chemotherapy alone with chemotherapy plus immunotherapy is emphasized.

### MATERIALS AND METHODS

*Patients studied.*—This study was commenced in April 1973. All the patients subjected to the treatment protocol had biopsy proven disseminated malignant melanoma. Because of extensive metastatic disease, all the patients were considered unsuitable for surgical management and some of them had previously been treated with irradiation and/or chemotherapy. Brief clinical details are presented in the Table and indicate the site of original primary, length of history and previous management. No patients were started on the treatment protocol within 8 days of any surgical intervention such as diagnostic biopsy. Before treatment, the patients were investigated to determine the extent of their metastatic disease. Routine biochemical and haematological screening was performed, the urine was examined for the presence of premelanogen; chest x-rays, skeletal surveys, liver function tests, liver scans and ultrasonograms were performed in all cases. Where indicated lymphangiography and bone marrow aspirations were carried out. Detailed mapping and measurements of all clinically detectable disease were performed.

Histological confirmation of the diagnosis was made in all cases but so far examination of the original primary tumours and appropriate grading and classification studies are incomplete.

*Immunization procedure.*—Melanoma cells obtained as previously described (Currie, Lejeune and Fairley, 1971), were withdrawn from a liquid nitrogen bank, rapidly thawed at 37 °C and washed 3 times in medium 199. They were then counted in a haemacytometer and diluted to give $2 \times 10^7$ cells in 0·9 ml of medium 199. After irradiation (10 Krad in a $^{60}$Co source) BCG was added.

Percutaneous BCG (Glaxo) was reconstituted and diluted in streptomycin-free medium to a concentration of 500 $\mu$g/ml; 0·1 ml of this suspension was then added to the 0·9 ml of tumour cells. This dose of BCG contained approximately $2 \times 10^6$ live organisms. This 1 ml of cells and BCG were then inoculated intradermally in 8 distinct sites in one limb at a time. Inoculations were made only into limbs that were macroscopically free of tumour. Each patient in the final protocol received allogeneic cells. For repeated immunizations a different batch of donor cells was employed each time (*i.e.* derived from different donor patients). This was done to maximize any possible helper effect due to histocompatibility antigens on the donor cells and to minimize any possible interference by the development of high titre anti-HLA antibodies which might abrogate any such " help ".

*Chemotherapy.*—5(3,3-dimethyl-triazeno)-imidazole-4-carboxamide (N.S.C. 45388) was administered intravenously at a dose of 2·5 mg/kg body weight for 5 consecutive days. On the first day the patients also received a single intravenous injection of vincristine at a dose of 1·4 mg/m$^2$. Antiemetic drugs were also given to minimize nausea and vomiting.

This chemotherapy regimen is remarkably non-toxic. In no patients did sufficient marrow toxicity occur to necessitate changes in the protocol. Very mild peripheral neuropathy, due no doubt to the vincristine, occurred in 2 patients but did not constitute a major problem. In one patient (ME360) the chemotherapy was stopped after 2 courses because she found the nausea distressing. In one further case (ME347) no chemotherapy was given as the patient lived too far away and refused admission to hospital at monthly intervals to receive chemotherapy. The results of this case are included in the Table because this patient showed a dramatic regression, due apparently to immunotherapy alone.

In the week following chemotherapy, occasional patients complained of lassitude, but on the whole the protocol was exceptionally well tolerated.

*Combined immunotherapy – chemotherapy protocol.*—In all these studies the patients started treatment with the immunization procedure. There is no rationale for this other than the fact that the patients could be investigated without the influence of prior chemotherapy affecting their immunological reactivity.

The major variable in a combined regimen such as this is the time interval between treatments. Currie and Bagshawe (1970) have shown in an animal model that the interval between chemotherapy and any subsequent immunotherapy is of crucial importance. In that particular study an interval of 12–14 days was found to be optimal. In different test systems, however, the intervals needed for an optimal effect may vary. This may well depend on both the tumour studied and the chemotherapeutic agent employed.

The final protocol chosen for the pilot study, based on the animal data of Currie and Bagshawe (1970) and the known

FIG. 1.—Combined immunotherapy–chemotherapy protocol employed in this study.

immunosuppressive properties of the chemotherapy, is illustrated in Fig. 1. Starting with immunotherapy there was a delay of 14 days, followed by 5 days chemotherapy. The next cycle commenced with immunotherapy given 14 days from the end of the previous course of chemotherapy. The treatment continued, where possible, for 6 cycles and the chemotherapy was then omitted and the immunotherapy continued at 28-day intervals.

*Assays of circulating soluble melanoma-specific antigen.*—Where technically feasible, patients were studied by the *in vitro* assay previously described which employed short-term cultures of target cells (Currie and Basham, 1972; Currie, 1973*b*).

Sera were obtained at frequent intervals before and after treatment and stored at − 20°C before testing. They were tested for specific inhibitory activity by incorporation at 5% into the lymphocyte suspensions before seeding onto melanoma target cells. The results were expressed as an inhibitory index which was derived from the crude cell counts thus:

$$\text{Inhibitory index} = \frac{\text{No. of cells killed in 5\% AB serum} - \text{No. of cells killed in 5\% patient's serum}}{\text{No. of cells killed in 5\% AB serum}} \times 100$$

where

No. of cells killed = mean cell number in serum only wells. − mean cell number in lymphocyte + serum wells

Specificity of the inhibitory effect was checked routinely by incorporating the test sera into lymphocyte–target cell combinations from patients with renal parenchymal cell carcinoma and results are quoted only from assays in which no nonspecific effects were detectable.

### RESULTS

### *Effects of immunization with irradiated allogeneic melanoma cells*

Previous studies (Currie, 1973*b*) have shown that active immunization with irradiated autologous tumour cells leads to a fall in the serum levels of circulating soluble antigen, present either free or bound to antibody. In that the cytotoxicity of peripheral blood lymphocytes from malignant melanoma patients showed a pattern of histogenic cross-reaction (Currie and Basham, 1972), it was decided to assess the effects of immunization of these patients with irradiated allogeneic melanoma cells. Figure 2 shows the results obtained in 5 patients with disseminated melanoma. In each case there was a dramatic, rapid but transient fall in circulating serum inhibitory activity at least as good as that previously obtained with autologous cells (Currie, 1973*b*). As the use of allogeneic cells made the immunization procedure practicable in all the patients, it was adapted as the standard immunization method in the current protocol.

### *Effect of admixing BCG with the tumour cells*

It was previously alleged that large numbers of inoculated tumour cells were

FIG. 2.—Effect of immunization (intradermally) with irradiated allogeneic melanoma cells on serum levels of specific inhibitory activity in 5 patients with disseminated malignant melanoma. The arrow denotes the time of injection.

needed to evoke a detectable *in vitro* effect (Ikonopisov *et al.*, 1970; Currie *et al.*, 1971). It was found that the incorporation of BCG into the cell suspension and the use of the intradermal route allowed the use of low numbers of cells for immunization, $2 \times 10^7$ allogeneic cells plus 50 $\mu$g BCG, giving a predictable fall in serum inhibitory activity (circulating antigen). This is in accord with the findings of Sokal, Aungst and Han (1972) who found that delayed hypersensitivity reactions to human tumour cell lines could be readily evoked using a similar immunization procedure.

What is the effect of BCG alone? In the case illustrated (Fig. 3) one of 3 patients so tested was first given intradermal BCG only, followed later by a mixture of BCG and allogeneic melanoma cells. It can be seen that the BCG alone had no detectable effect on the serum inhibitory activity level, whereas the inclusion of tumour cells in the mixture led to a prompt fall. This was the result in all cases tested in this way. It seems probable that the BCG given in this manner is acting as a local adjuvant rather than a nonspecific systemic " booster " of immunological reactivity, although further studies will be needed to elucidate the precise role of the BCG.

*Effect of the immunotherapy—chemotherapy protocol on serum antigen levels*

The first case treated with this combined protocol (ME338) had serial measurements of specific serum inhibitory activity made. Details of this case are presented later in this paper. However, the impetus for carrying out this pilot

FIG. 3.—Serum inhibitor levels in a patient (ME347) treated first with intradermal BCG only and then with BCG plus allogeneic melanoma cells. BCG alone had no effect on the serum inhibitory activity whereas the addition of tumour cells led to a significant fall.

FIG. 4.—Serial estimations of circulating " melanoma specific antigen " in ME338 during the first 3 treatment courses. The stepwise decline in serum activity was associated with regression of his massive retroperitoneal tumour.

study was provided by the fact that his massive intra-abdominal tumour underwent a dramatic regression while the *in vitro* studies were being performed. The serial levels of serum inhibitor in this case are illustrated in Fig. 4. Following the first immunization there was a rapid decline in serum inhibitor levels. This recovered quite rapidly and was apparently unaffected by the chemotherapy; however, after the next immunization there was an even greater and more sustained fall in inhibitor levels in the serum. After the third cycle of treatment the level had fallen to zero. This sequence of events occurred concurrently with the disappearance of his massive tumour, although in this first case the timing schedule was not strictly adhered to. The levels of inhibitor remained at

Fig. 5.—Serum inhibitor levels in 2 patients (ME352 and ME360) with skin metastases during the first 2 courses of treatment. The prompt fall in inhibitory activity occurred concurrently with tumour regression in both cases.

Fig. 6.—Two cases (ME347 and ME362) with massive hepatic involvement. ME347 underwent a temporary regression whereas the disease in ME362 progressed inexorably. The serial measurements of inhibitory soluble antigen correlated with the clinical picture in each case.

zero until his eventual death from intestinal obstruction.

In Fig. 5 and 6, 4 more cases are illustrated throughout the first 2 cycles of treatment. Figure 5 shows the results obtained in 2 women with disease confined to the subcutaneous tissues, although in one (ME352) it was widely disseminated. In both cases the first cycle of treatment led to a profound and sustained fall in inhibitor levels. Objective regression (complete in ME352 and partial in ME360) occurred at the same time. In Case no. ME360, the partial regression (at least 50% of all measurable disease) was maintained for 11 months, whereas the total regression in ME352 was maintained 9 months before recurrence of a single subcutaneous nodule. Moreover, the patient with maintained partial remission (ME360) had received only 2 courses of chemotherapy. In both cases inhibitory activity remained undetectable over the first 6 months.

In Fig. 6, 2 patients are illustrated who presented with much more advanced disease. Both previously had primary retinal tumours treated surgically and had both presented with subsequent massive liver involvement. Case no. ME345 survived for 13 months after starting treatment and during that time objective regression, as indicated by shrinking of both liver size and of a large palpable epigastric nodule, occurred and lasted approximately 2–3 months. In Case no. ME362 the liver was, at presentation, massively involved and was quite unaffected by the treatment. Subsequent intrahepatic infusion of DTIC gave subjective relief of liver pain but no evidence of objective regression was obtained and she subsequently died 14 weeks after presentation.

There is, as can be seen from Fig. 6, a striking correlation between the *in vitro* assay results and the clinical outcome. In ME362 the levels of circulating inhibitor were high and rose progressively, being virtually unaffected by the treatment, whereas ME345 showed a fall in inhibitory activity after the first 2 immunizations.

The results obtained from the *in vitro* assays seem to indicate that serial assays of circulating tumour specific inhibitor correlate with the clinical outcome of the combined immunotherapy–chemotherapy regimen. Furthermore, they support the notion that the immunization procedure is capable of affecting the patient's own tumour directed immunological reactions in a meaningful manner.

### *Clinical results*

The definition of objective regression in malignant melanoma is difficult. By convention such a regression is often defined as a 50% reduction in the diameter of measurable lesions lasting at least 30 days and in the absence of disease progression elsewhere. Lack of progression of the disease, alone, although possibly an important therapeutic effect is much more difficult to assess. In order to avoid some of the semantic problems associated with such definitions, brief details of the cases treated in this study are shown in the Table, and some of those undergoing objective regression are described in some detail.

*ME338.*—In 1967 this man, then aged 36, noticed an area of pigmented skin on his anterior abdominal wall which was spreading rapidly and eventually started to bleed. This lesion was widely excised and was histologically diagnosed as malignant melanoma. At the same time an involved lymph node was removed from his left axilla. After an interval of 6 months another involved lymph node became palpable in the right axilla and this was also excised. One month later a local recurrence was excised from the original primary site. In the following 2 years he developed several subcutaneous metastases on the chest wall, abdomen and right arm and these were treated with irradiation with some success. Later in 1972, however, his general health deteriorated and he was found to have severe anaemia. This was found to be due to bleeding from the gut and he was managed by periodic blood transfusions. He was referred to this centre complaining of abdominal discomfort and distention. On examination and after detailed investigation, he was found to have a large intra-abdominal mass involving the left para-aortic lymph nodes (by lymphangiography) and a grossly abnormal liver scan. At laparotomy he was found to have a massive retroperitoneal tumour. A superficial lobe of this tumour was invading both duodenum and jejunum and this part of the tumour and involved gut were excised, leaving behind the larger deeper lobe of the tumour. Histologically the mass excised was found to consist of poorly differentiated amelanotic melanoma. He made an excellent recovery from this surgical intervention and subsequently on palpation of the abdomen a fixed mass of approximately 15 cm in diameter could be felt lying to the left of the midline at the level of the umbilicus.

In May 1973 he was immunized with irradiated tumour cells and BCG and started on the combination treatment. During each course of chemotherapy it was noticed in this case that the immunization sites flared up with induration, erythema and tenderness, subsiding again after the cessation of chemotherapy. Over the first 3 months of treatment the palpable abdominal lesion became much smaller in diameter, shrinking to approximately 5 cm in diameter. However, despite this excellent progress he eventually ($4\frac{1}{2}$ months after starting treatment) had several grand mal epileptic seizures. Despite negative investigations for cerebral deposits, he was treated with cranial irradiation. Approximately 1 month later he suddenly became extremely ill, developing abdominal obstruction and died. At autopsy the only detectable tumour was the retroperitoneal lesion to which many loops of bowel adhered; his liver, para-aortic lymph nodes and brain were free of disease and the tumour mass consisted of necrotic debris with a small rim of viable tumour.

*ME365.*—In 1968 this lady, aged 39, noticed that a mole on her left thigh was enlarging and, being a nurse, she asked for it to be removed. Histologically this lesion was reported to be benign. However, 4 years later she developed a pea-size nodule under the excision scar and this was widely excised and found to be a malignant melanoma. Six months later she developed a mass in her left groin and on investigation there was evidence of widespread disease. Apart from the mass of nodes in the left groin, lymphangiography demonstrated massive intrapelvic node involvement on the left side. There were several large lung metastases visible on chest x-ray, and a liver scan showed several cold areas in the right lobe of the liver.

She was therefore started on immunotherapy and chemotherapy. Within 3 months the mass in the left groin had regressed completely and the pelvic mass was much smaller. After a further 3 months, at which time the chemotherapy was stopped, the liver scan was reported as normal, the cold areas having resolved, and the only detectable abnormality at that time was a single small nodule in the lower lobe of her right lung and a small pelvic node on the left side. She continued on monthly immunotherapy and was given a low dose of local irradiation to the lesion in the right lung. Both these small residual lesions, in lung and pelvis, have remained static now for 5 months. At 11 months after starting treatment she remains fit and well with no evidence of disease elsewhere.

*ME352.*—This lady, aged 51, presented in 1970 with a primary malignant melanoma on her left calf. This was widely excised and the site skin grafted. She remained well for 2 years until a local subcutaneous recurrence occurred in her left lower leg. At the same time she developed acute appendicitis. At appendicectomy no evidence of intra-abdominal metastasis was found. The local lesion on her leg was excised. However, 6 months later multiple subcutaneous nodules developed on her left leg and these were treated with intra-arterial melphelan. This had no effect and so most of the lesions were surgically excised. Another 6 months later she presented with further deposits on the leg but now there were palpable metastases in the scalp, on the left shoulder and in the left breast. The lesion in her breast and most of those on the leg were excised. Histologically they were all shown to be deposits of malignant melanoma. The subcutaneous lesions on the scalp, on the shoulder and those remaining on the leg were left alone. There was no evidence of visceral involvement from clinical examination or from the investigations performed. She was therefore started on the combination treatment protocol.

After 3 months treatment, all the nodules regressed completely and she remained well for a further 7 months. However, 10 months after starting the treatment local recurrence occurred in the left leg and this was surgically excised. In the 2 weeks following surgery there was a rapid, almost explosive, growth of multiple subcutaneous lesions on the left leg with involvement of lymph nodes in the left groin. She had by this time been off chemotherapy for 4 months but had continued with monthly immunizations; she was therefore started on DTIC and vincristine again and this produced a transient regression of her recurrent disease which was maintained for a further 2 months.

*ME382.*—This patient, a lady aged 44, originally presented in 1960 with a pigmented lesion on her left calf; it had been there for many years but in the previous few weeks had grown larger and had occasionally bled. This was excised surgically and on histological examination was described as a " junctional naevus with a central nodule showing very early malignant change ". She remained well for a further 12 years after this episode, until discovering a subcutaneous nodule in the left thigh. This was excised and on histological examination shown to be overt malignant melanoma. Six months later the inguinal lymph nodes on the left side became enlarged and she underwent block dissection of the left groin. Two of the excised nodes were found to be involved, the uninvolved nodes showing marked reactive hyperplasia. Six months later yet another nodule developed in her left thigh. This was excised and histologically was " malignant melanoma consisting of plump spindle cells with abundant mitotic figures ". After yet another 6 months, 2 small nodules of malignant melanoma were excised from her scalp and this time a chest x-ray revealed the presence of multiple lung metastases.

On examination, she was a healthy looking woman with no evidence of subcutaneous disease or other clinically detectable manifestations of metastatic melanoma. On investigation, the only detectable disease was confined to the chest.

She was therefore started on the current immunotherapy–chemotherapy protocol with minor modifications of the chemotherapy. No vincristine was given and the dose of DTIC employed was 250 mg/m$^2$. She tolerated the treatment well, not even requiring hospitalization for the chemotherapy. After 3 months treatment, the chest x-ray showed evidence of regression of the metastatic lesions. No further disease has recurred in other sites and she remains extremely fit after a further 6 months.

Of the 30 cases treated in this pilot study, objective regression of disease (partial or complete) occurred in a total of 17 (*i.e.* 57%). In that the regression rate to be anticipated from the use of chemotherapy alone is in the order of 20%, we believe that the active immunotherapy may have a significant adjuvant effect. We are therefore encouraged to embark on the appropriate controlled trial of immunotherapy plus chemotherapy *vs* chemotherapy alone.

### DISCUSSION

The systemic deployment of immunological responses for the treatment of cancer patients is once again under investigation in many centres. However, most controlled studies of immunotherapy have so far provided remarkably little evidence of clinical benefit. For instance, active immunotherapy using autologous irradiated tumour cells has been shown to be without effect in patients with glioblastoma multiforme when used as an adjunct to radiotherapy in a randomized controlled trial (Bloom *et al.*, 1973). Furthermore, nonspecific active treatment with BCG, despite intense investigation, has been shown to be ineffective in maintaining remission in acute lymphoblastic leukaemia (Heyn *et al.*, 1973; MRC, 1971) and in Burkitt's lymphoma (Ziegler and Magrath, 1973). However, Sokal (1973) has claimed, using historical controls, that immunotherapy can be beneficial in chronic myeloid leukaemia. Furthermore, also employing historical controls, Gutterman and his co-workers (1973) have alleged that immunotherapy using BCG is of value in the treatment of malignant melanoma. The use of both irradiated blast cells and BCG has been shown to prolong remission length and survival in patients with acute myelogenous leukaemia previously brought into haematological remission with chemotherapy (Powles *et al.*, 1973). These effects, obtained in a controlled trial, indicate that under some circumstances an immunological procedure may significantly influence the course of a malignant disease. Although the results of this study do not constitute a major advance in the treatment of acute myelogenous leukaemia, in that the patients still die of the disease, it is so far one of the few controlled trials which clearly demonstrate an effect due to immunotherapy. Vogler and Chan (1974) have recently described a similar prolongation in remission in the same disease using BCG alone as the immunotherapy. Such studies seem to illustrate the potential value of appropriate combinations of immunotherapy with other treatment methods.

In experimental animals such an adjuvant activity of immunotherapy has been described by Haddow and Alexander (1964). Currie and Bagshawe (1970) have described the successful combination of chemotherapy with a form of immunotherapy and the value of this sort of combination has been confirmed in experimental animals by other workers (Pearson *et al.*, 1972). However, the combination of an immunization procedure with a potentially immunosuppressive form of chemotherapy will obviously be complex. The current enthusiasm for giving chemotherapy intermittently in high doses could perhaps permit the inclusion of an immunization procedure between courses. Cheema and Hersh (1971) have investigated some of the effects of chemotherapy on lymphoid cell function. Many agents given in a single large dose lead to a severe depression of lymphoid cell function which does, however, recover, often with rebound, after 8–10 days. DTIC has little immunosuppressive activity (Bruckner, Mokyr and Mitchell, 1974) and the kinetics of any effect it might have on lymphoid cells is unclear. As DTIC is currently the drug of choice in the treatment of disseminated malignant melanoma, the optimal immunization technique already described was incorporated into a cyclical combination with this drug.

The immunization technique employed in this study was extremely well tolerated. Local reactions at the injection sites were, however, variable. In the majority of patients the local lesions produced rarely caused inconvenience or pain, were usually less than 1 cm in diameter and were not associated with any overt systemic disturbances such as fever, malaise or impaired liver function. After several cycles of treatment, the reactions did become more severe in several patients. After 3 or 4 monthly immunizations the dose of BCG used was halved in those patients whose previous injection site reactions were over 1 cm in diameter. In such hyper-reactive patients, inoculations of allogeneic melanoma cells alone frequently evoked vigorous delayed hypersensitivity reactions. In one case (ME 352) inoculation of autologous tumour cells evoked such a reaction. Unfortunately, autologous cells were not available in most of the cases treated. Where possible, immunization was confined to the upper limbs because inoculation intradermally into the thigh region, especially in women, occasionally led to ulceration, due apparently to local fat necrosis.

It was noticed that in those patients undergoing tumour regression the local immunization site lesions were often more aggressive. In some patients there was little or no reaction around the injection sites; in none of these did tumour regression occur. This finding suggested that the ability of the patients to mount a delayed hypersensitivity reaction may be important in determining the effects of the treatment. An attempt to examine this possibility gave somewhat enigmatic results. Eight of the patients were sensitized to dinitrochlorobenzene

(DNCB) and then skin tested 14 days later. Three patients were completely unreactive to any test concentration of DNCB whereas the other 5 all gave normal reactions. The 3 negative DNCB cases, however, all produced dramatic local reactions to the BCG–tumour cell innoculum and all 3 underwent objective tumour regression (ME352, ME356, ME-381). One of the DNCB positive cases gave a very brisk delayed hypersensitivity reaction even to the lowest test concentration and yet failed to produce local responses to the BCG and tumour cell mixture or to show any sign of tumour regression. In other words, DNCB skin reactivity did not predict eventual response to the immunization schedule. Whether or not DNCB skin reactivity constitutes a meaningful assay of delayed hypersensitivity reactions in general remains unclear at present.

The number of objective regressions occurring in this small pilot study was surprisingly high. Previous studies at this centre using chemotherapy of various types (including DTIC) have provided a very low incidence of objective regressions. Using DTIC, our experience has not matched that of many other centres—of the last 16 cases so treated regression occurred in only one.

Luce and his colleagues (Luce *et al.*, 1970) reported a clinical study in which DTIC was used in 110 patients with disseminated malignant melanoma. Objective anti-tumour effects were seen in 21 of these (*i.e.* 19%). In a small study of 19 cases, Ahmann and his colleagues (Ahmann, Hahn and Bisel, 1974), combined DTIC with vincristine in a manner similar to our present chemotherapy regimen, and described objective responses in only 4 (*i.e.* 21%). Luce (1972), in reporting the results of the South West Cancer Chemotherapy study group, showed that objective regression was obtained in 26% of women but only in 13% of men treated with DTIC. He concluded that DTIC " has proven, in extensive trials, to be the most effective single agent yet developed ". Combination with other agents has so far provided little or no added advantage over the use of DTIC alone. Luce (1972), describing pooled data from several trials of DTIC, noted that out of a total series of 733 cases objective regression was obtained in 169 (23%).

There are some clues from this study that the immunotherapy procedure may well be playing an important role. The correlation between clinical effects and *in vitro* assay results, although not studied in all the cases, is nevertheless striking. Furthermore, the regression obtained in the one patient (ME347) treated with immunotherapy alone is highly suggestive and, taken with the strikingly high incidence of objective regression occurring in this group, compared with our own historical controls or with other published studies of patients treated with chemotherapy alone, argues in favour of the immunotherapy acting as an important adjunct to chemotherapy.

Objective regression, however defined, may not necessarily have much clinical relevance. It does, however, provide a visible manifestation of anti-tumour activity and therefore allows an assessment of a treatment protocol at an early stage. The effects of active immunotherapy plus chemotherapy on the overall survival (as well as regression rate) of patients will obviously have to be assessed in some sort of controlled clinical comparison. The relative effectiveness of immunotherapy as an adjunct to chemotherapy in the treatment of disseminated malignant melanoma can only be ascertained by the use of such concurrent controlled trials. Furthermore, evidence obtained from this study should allow the design of rational combinations of immunotherapy and chemotherapy in situations of greater clinical relevance such as the treatment of high risk primary tumours and in the prophylaxis of recurrence following surgery.

This study illustrates our approach to the development and clinical application of a form of so-called immunotherapy. We believe that the essential features of such an approach must include assays of the effects of the immunological manipulation employed on some aspect of tumour specific host reactivity. At present an *in vitro* assay for the detection of circulating soluble tumour specific inhibitory factors seems to be the most appropriate test system for determining the most effective method of immunization and allowing its subsequent incorporation into a combined regimen with chemotherapy.

Studies in these laboratories are supported by grants made to the Chester Beatty Research Institute by the Cancer Research Campaign and the Medical Research Council. G. A. Currie thanks the Ludwig Institute for Cancer Research for financial support.

We thank our clinical colleagues at the Royal Marsden Hospital for their co-operation in carrying out this study. We acknowledge with gratitude help from the surgeons who referred many of these cases to us. These include Mr C. I. Cooling, Mr W. H. W. Jayne and Mr J. M. Edwards. Case ME382 was treated in collaboration with Dr W. F. White at St Luke's Hospital, Guildford, Surrey.

### REFERENCES

AHMANN, D. L., HAHN, R. G. & BISEL, H. F. (1974) Evaluation of 1(2-Chloroethyl-3-4-methyl cyclo hexyl)-1-Nitrosourea (Methyl CCNU, NSC 95441) *vs* Combined Imidazole Carboxamide (NSC 45388) and Vincristine (NSC 67574) in Palliation of Disseminated Malignant Melanoma. *Cancer, N.Y.*, **33**, 615.

BLOOM, H. J. G., PECKHAM, M. J., RICHARDSON, A. E., ALEXANDER, P. & PAYNE, P. M. (1973) Glioblastoma Multiforme: a Controlled Trial to Assess the Value of Specific Active Immunotherapy in Patients Treated with Radical Surgery and Radiotherapy. *Br. J. Cancer*, **27**, 253.

BRUCKNER, H. W., MOKYR, M. B. & MITCHELL, M. S. (1974) Effect of Imidazole-4-carboxamide 5(3,3-Dimethyl-1-triazeno) on Immunity in Patients with Malignant Melanoma. *Cancer Res.*, **34**, 181.

CHEEMA, A. R. & HERSH, E. M. (1971) Patient Survival after Chemotherapy and its Relationship to *in vitro* Lymphocyte Blastogenesis. *Cancer, N.Y.*, **28**, 851.

CURRIE, G. A. (1973*a*) Circulating Antigen as an Inhibitor of Tumour Immunity in Man. In *Immunology of Malignancy*, ed. M. Moore, N. W. Nisbet and Mary V. Haigh. *Br. J. Cancer*, **28**, Suppl. I, 153.

CURRIE, G. A. (1973*b*) Effect of Active Immunization with Irradiated Tumour Cells on Specific Serum Inhibitors of Cell-mediated Immunity in Patients with Disseminated Cancer. *Br. J. Cancer*, **28**, 25.

CURRIE, G. A. & BAGSHAWE, K. D. (1970) Active Immunotherapy with *Corynebacterium parvum* and Chemotherapy in Murine Fibrosarcomas. *Br. med. J.*, i, 541.

CURRIE, G. A. & BASHAM, C. (1972) Serum Mediated Inhibition of the Immunological Reactions of the Patient to His Own Tumour: a Possible role for Circulating Antigen. *Br. J. Cancer* **26**, 427.

CURRIE, G. A., LEJEUNE, F. & FAIRLEY, G. H. (1971) Immunisation with Irradiation Tumour Cells and Specific Lymphocyte Cytotoxicity in Malignant Melanoma. *Br. med. J.*, ii, 205.

GUTTERMAN, J. V., MAVLIGIT, G. & MCBRIDE, C. (1973) Active Immunotherapy with B.C.G. for Recurrent Malignant Melanoma. *Lancet*, i, 1208.

HADDOW, A. & ALEXANDER, P. (1964) An Immunological Method of Increasing the Sensitivity of Primary Sarcomas to Local Irradiation with X-rays. *Lancet*, i, 452.

HEYN, R., BORGES, W. & JOO, P. (1973) B.C.G. in the Treatment of Acute Lymphocytic Leukemia (A.L.L.). *Proc. Am. Ass., Cancer Res.*, **14**, 45.

IKONOPISOV, R. L. (1970) Auto-immunisation with Irradiated Tumour Cells in Human Malignant Melanoma. *Br. med. J.*, ii, 752.

LUCE, J. K., THURMAN, W. G., ISAACS, B. L. & TALLEY, R. W. (1970) Clinical Trial with the Anti-tumour Agent 5(3,3-Dimethyl-1-triazeno)-imidazole-4 carboxamide (NSC–45388). *Cancer chemother. Rep.*, **54**, 119.

LUCE, J. K. (1972) Chemotherapy of Malignant Melanoma. *Cancer, N.Y.*, **30**, 1604.

MEDICAL RESEARCH COUNCIL (1971) Treatment of Acute Lymphoblastic Leukaemia: Comparison of Immunotherapy (BCG), Intermittent Methotrexate and No Therapy after a Five Month Intensive Cytotoxic Regimen (Concord Trial). *Br. med. J.*, iv, 189.

PEARSON, J. W., PEARSON, G. R., GIBSON, W. T., CHERMANN, J. C. & CHIRIGOS, M. A. (1972) Combined Immunostimulation Therapy against Murine Leukemia. *Cancer Res.*, **32**, 904.

POWLES, R. L., CROWTHER, P. & BATEMAN, C. J. T. (1973) Immunotherapy for Acute Myelogenous Leukaemia. *Br. J. Cancer*, **28**, 365.

SOKAL, J. E. (1973) Immunologic Studies in Lymphoma and Leukemia and Exploration of Antitumor Immunotherapy. *J. surg. Oncol.*, **5**, 557.

SOKAL, J. E., AUNGST, C. W. & HAN, T. (1972) Use of BCG as Adjuvant in Human Cell Vaccines. *Cancer Res.*, **32**, 1584.

VOGLER, W. R. & CHAN, Y-K. (1974) Prolonging Remission in Myeloblastic Leukaemia by Tice-strain Bacillus Calmette-Guérin. *Lancet*, ii, 128.

ZIEGLER, J. L. & MAGRATH, I. T. (1973) BCG Immunotherapy in Burkitt's Lymphoma: Preliminary Results of a Randomized Clinical Trial. *Natn. Cancer Inst. Monog.*, **39**, 199.